# Acridine-Based Chalcone 1C and ABC Transporters

**DOI:** 10.3390/ijms26094138

**Published:** 2025-04-27

**Authors:** Ondrej Franko, Martina Čižmáriková, Martin Kello, Radka Michalková, Olga Wesołowska, Kamila Środa-Pomianek, Sérgio M. Marques, David Bednář, Viktória Háziková, Tomáš Ján Liška, Viera Habalová

**Affiliations:** 1 Department of Pharmacology, Faculty of Medicine, Pavol Jozef Šafárik University, 040 11 Košice, Slovakia; 2 Department of Biophysics and Neurobiology, Wroclaw Medical University, 50-369 Wrocław, Poland; 3 Loschmidt Laboratories, Department of Experimental Biology and RECETOX, Faculty of Science, Masaryk University, 625 00 Brno, Czech Republic; 4 International Clinical Research Center, St. Anne’s University Hospital, 656 91 Brno, Czech Republic; 5 Institute of Chemistry, Faculty of Science, Pavol Jozef Šafárik University, 040 11 Košice, Slovakia; 6 Department of Medical Biology, Faculty of Medicine, Pavol Jozef Šafárik University, 040 11 Košice, Slovakia

**Keywords:** chalcone, drug efflux, transporter, multidrug resistance, expression, ABCB1, ABCC1, ABCG2, colorectal carcinoma, galectin-1

## Abstract

Chalcones, potential anticancer agents, have shown promise in the suppression of multidrug resistance due to the inhibition of drug efflux driven by certain adenosine triphosphate (ATP)-binding cassette (ABC) transporters. The gene and protein expression of chosen ABC transporters (multidrug resistance protein 1, ABCB1; multidrug resistance-associated protein 1, ABCC1; and breast cancer resistance protein, ABCG2) in human colorectal cancer cells (COLO 205 and COLO 320, which overexpress active ABCB1) was mainly studied in this work under the influence of a novel synthetic acridine-based chalcone, 1C. While gene expression dropped just at 24 h, compound 1C selectively suppressed colorectal cancer cell growth and greatly lowered ABCB1 protein levels in COLO 320 cells at 24, 48, and 72 h. It also reduced ABCC1 protein levels after 48 h. Molecular docking and ATPase tests show that 1C probably acts as an allosteric modulator of ABCB1. It also lowered galectin-1 (GAL1) expression in COLO 205 cells at 24 h. Functional tests on COLO cells revealed ABCB1 and ABCC1/2 to be major contributors to multidrug resistance in both. Overall, 1C transiently lowered GAL1 in COLO 205 while affecting important functional ABC transporters, mostly ABCB1 and to a lesser extent ABCC1 in COLO 320 cells. COLO 320’s absence of GAL1 expression points to a possible yet unknown interaction between GAL1 and ABCB1.

## 1. Introduction

Colorectal cancer represents a significant global health burden and is among the most lethal gastrointestinal malignancies. GLOBOCAN statistics show that in 2022, there were over 1.9 million new cases recorded and more than 904,000 deaths connected to this condition. Among all cancers worldwide, colorectal cancer ranks second in death (9.3%) and third in incidence (9.6%) [1]. Projections suggest that by 2035, cases of rectal and colon cancer will increase by 60.0% and 71.5%, respectively [2]. Late-stage diagnosis, which limits treatment options and lowers survival rates, is a major cause of the poor prognosis of colorectal cancer. Usually, the conventional method for treating advanced colorectal cancer cells is through cytoreductive surgery followed by chemotherapy using drugs including 5-fluorouracil, capecitabine, irinotecan, and oxaliplatin [3]. Treatment regimens have also included new treatment strategies such antiangiogenic therapy, immunotherapy, targeted therapies, and adaptive radiotherapy [4,5]. Still, because of ongoing treatment resistance, death rates stay high even with these developments.

Multidrug resistance (MDR), a phenomenon in which cancer cells become resistant to several chemotherapeutic drugs, is significantly influenced by drug efflux mediated by membrane adenosine triphosphate (ATP)-binding cassette (ABC) transporters [6,7,8,9]. This feature was observed in various malignancies, including colorectal cancer [10]. Genetic and epigenetic changes, hormonal control, and the activation of signaling pathways such as Wnt/β-catenin and MAPK/ERK are among the factors that most often cause these transporters to be overexpressed in cancer cells [11,12,13]. Tumor cells can actively expel chemotherapeutic drugs thanks to this upregulation, therefore lowering intracellular drug levels and compromising treatment efficacy and finally escaping cytotoxic consequences.

Based on the sequence homology of nucleotide-binding domains (NBDs) and trans-membrane domains (TMDs), gene structure, and domain organization [14], the human ABC protein family is divided into seven subfamilies: ABCA to ABCG. Due to the absence of TMDs, members of subfamilies E and F are implicated in non-transport functions [15,16]. Based on TMD folding patterns, another classification divides human ABC transporters into type IV and type V subcategories [17]. Usually, TMDs enable substrate identification and transport, while NBDs control nucleotide binding and hydrolysis. Three important proteins from the ABC transporter family being particularly researched in relation to drug resistance are ABCB1 (multidrug resistance protein 1, MDR1 or P-glycoprotein), ABCC1 (multidrug resistance-associated protein 1, MRP1), and ABCG2 (breast cancer resistance protein, BCRP) [18,19,20].

Currently, there are no defined clinical tactics to properly offset ABC transporter-mediated chemoresistance, highlighting a vital treatment gap [21]. Recent studies have underlined the need for safe, selective inhibitors of ABC transporters. Interestingly, in vivo studies have suggested a new method whereby extended use of ABCB1 inhibitors during drug holidays greatly increases general survival [22]. By means unrelated to drug efflux suppression, these inhibitors could even stop the evolution of drug resistance. Such results point to a paradigm change from defeating drug resistance to preventing or postponing its onset.

From non-specific first-generation medications like verapamil to very selective third-generation chemicals like tariquidar, ABC transporter inhibitor development has advanced across four generations [8,23,24]. Emphasizing natural chemicals and their derivatives, the fourth generation provides better specificity and lower toxicity to fight multidrug resistance. Of these, flavonoids and chalcones have drawn great interest for their therapeutic possibilities [25,26]. Chalcones’ straightforward structural framework has drawn interest over the last ten years as it enables simple alterations to produce a wide spectrum of active analogs with anticancer qualities [27,28]. Many studies have investigated the molecular mechanisms behind the anticancer and antiproliferative effects of chalcones [27,29,30,31,32,33,34]. By lowering ABC transporter expression or activity, these chemicals have shown potential to reverse chemoresistance [35,36,37,38]. Studies from our department have also revealed that the synthetic acridine-based chalcone analog 1C selectively targets cancer cells [39] and displays different antiproliferative qualities [29,40]. Furthermore, it may function as a potent inhibitor of drug efflux. Human colorectal adenocarcinoma cells overexpressing the ABCB1 efflux pump [41] showed this effect. So far, though, the way this chemical affects ABCB1 efflux has not been studied.

Natural and synthetic chemicals can affect ABC transporters through various molecular mechanisms including competitive or non-competitive inhibition, membrane lipid bi-layer disruption, upstream regulator modulation, or signaling pathway alterations [42,43,44,45,46]. Recent research has uncovered the significance of increased galectin levels, specifically galectin-1 (GAL1) and galectin-3 (GAL3) in controlling ABCB1 transporter expression and fostering chemoresistance [47,48,49,50]. Curiously, chalcone-derived chemicals have been found to possibly inhibit GAL1 [51] and GAL3 [52]. However, the interplay between chalcone-derived compounds, galectins, and ABC transporters is still not well known, and more research is needed.

The objective of this experimental work is to examine the interaction of acridine-based chalcone analog 1C with the ABCB1 transporter molecule, as well as its effects on the protein and gene expression of this efflux pump in human colorectal adenocarcinoma cells. It also investigates the effects of the compound on the protein and gene expression of two other important ABC transporters, ABCC1 and ABCG2. Furthermore, the evaluation of chalcone effects on GAL1 protein expression in cancer cells is the next objective of this study. The work also expands the analysis of 1C’s antiproliferative effects to hitherto unexamined healthy cell lines, thus providing a better understanding of its selective action against tumor cells.

## 2. Results

### 2.1. Viability Assays

Preliminary viability assays were conducted to verify the acridine-based chalcone 1C’s activity and selectivity before proceeding with all following experiments.

**Figure 1:**
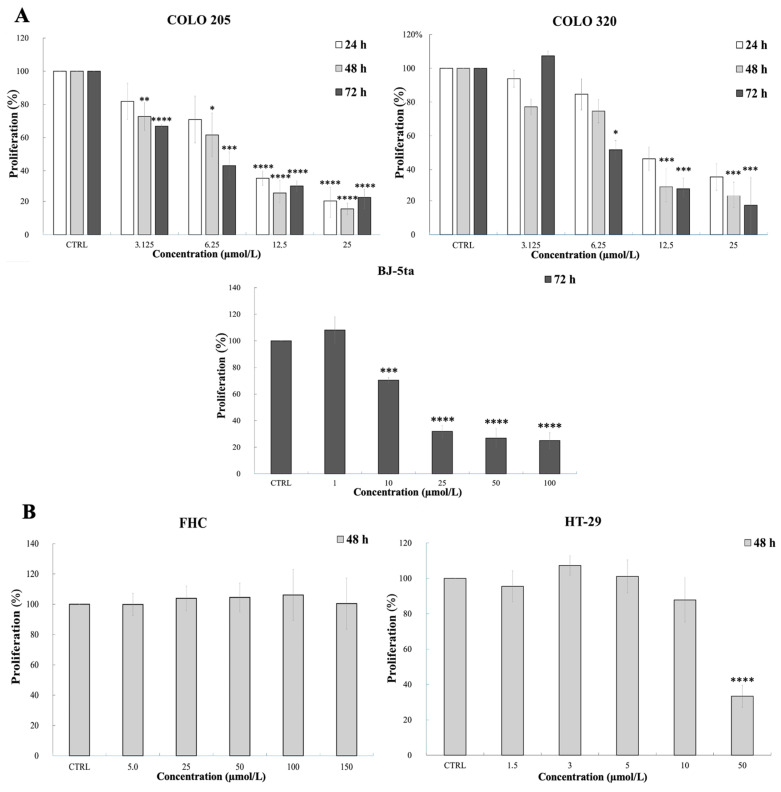
(**A**) Effect of 1C on the viability of COLO 205, COLO 320, and BJ-5ta, as determined by the MTT assay. (**B**) Effect of 1C on the viability of HT-29 and FHC cells, as determined by the SRB assay. Results are presented as the mean ± standard deviation from three independent experiments. Statistical significance: * *p* < 0.05, ** *p* < 0.01, *** *p* < 0.001, and **** *p* < 0.0001 for treated vs. the control.

The antiproliferative activity of compound 1C was assessed using the methylthiazoltetrazolium (MTT) colorimetric assay in cancerous (parental human colorectal adenocarcinoma cells, COLO 205, and human colorectal adenocarcinoma cells overexpressing ABCB1, COLO 320) and non-cancerous (human foreskin fibroblasts, BJ-5ta) cell lines, with the cells being treated with increasing concentrations of the compound. The viability assay with 1C on COLO cell lines have already performed in previous research [41]; the current tests were performed to validate the effects of a new batch of the substance. The testing on BJ-5ta was conducted for the first time to establish the selectivity of the drug. The concentrations of compound used, exposure times, cell survival percentages after incubation with 1C, and statistical comparisons are presented in Figure 1A and Tables S1–S3. As shown by the average half-maximal inhibitory concentration (IC_50_) values of tested cell lines (Table 1), 1C demonstrated significantly decreased inhibitory effects on the cell viability of the non-cancerous cell line (BJ-5ta) than on the tested cancer cells after 72 h of incubation. The selective effect of the substance on tumor cells, as opposed to healthy cells, is further supported by the selectivity index values (Table 1). COLO cell lines were selected for further experiments aimed at studying chalcone 1C in relation to ABC transporters.

The sulforhodamine B (SRB) assay was used to assess the impact of 1C on the growth of other human colorectal adenocarcinoma cells (HT-29) and human normal fetal colon epithelial cells (FHC). The data from this assay showed that the compound we tested had a concentration-dependent inhibitory effect on the cell viability of HT-29 cancer cells, while non-cancerous FHC cells exhibited no reduction in cell growth, even at high concentrations (Figure 1B and Tables S4 and S5). For cancer cell line HT-29, the corresponding average IC_50_ value was significantly decreased after 48 h with 1C in comparison with non-malignant cells (FHC), with the selectivity index approaching a value of four (Table 1).

Dimethyl sulfoxide (DMSO) did not show any inhibitory activity on cell viability at a concentration of less than 1% in all experiments.

To sum up, the results conducted using two different assays suggest that compound 1C effectively inhibits the viability of several colorectal cancer cells (COLO 205, COLO 320, and HT-29) while sparing non-cancerous cells (BJ-5ta and FHC), highlighting its potential as a selective anticancer agent.

### 2.2. Effect of 1C on ABCB1

#### 2.2.1. Chalcone 1C and ABCB1 Protein Expression

**Figure 2:**
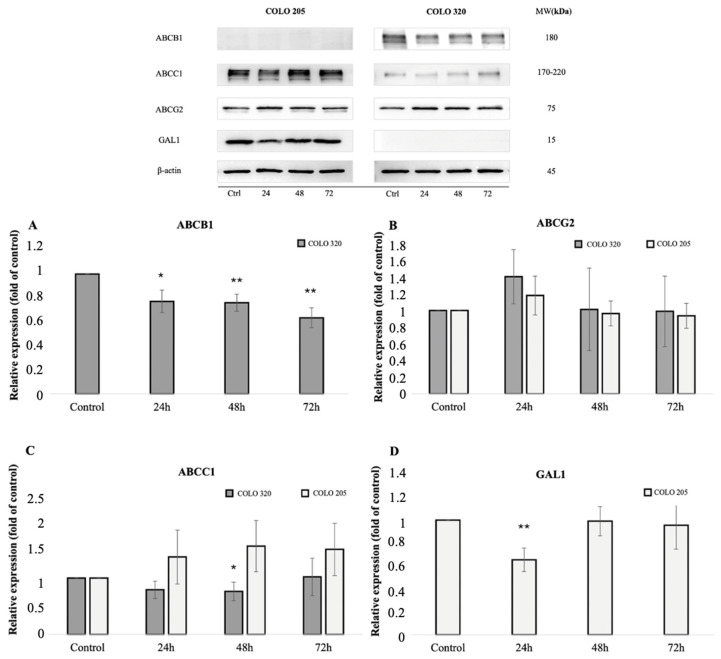
Western blot analysis of 1C effects on the protein expression of ABC transporters ((**A**) ABCB1, (**C**) ABCC1, and (**B**) ABCG2) and (**D**) galectin-1 (GAL1) after 12, 24, and 48 h of incubation. Graphs depicting a change in the expression of proteins in cells exposed to the 1C chalcone derivative. Significance levels are indicated as follows: * *p* < 0.05 and ** *p* < 0.01 in comparison to the DMSO-treated control group. β-actin was used as a loading control. ABCB1 was not detected in the COLO 205 cell line, and no GAL1 signals were observed in COLO 320 cells.

The protein expression levels of the ABCB1 transporter were evaluated in COLO 205 and COLO 320 cells using standard Western blot analysis (Figure 2).

Cells were treated with the 1C chalcone derivative at a concentration of 5 µmol/L for 24, 48, and 72 h. A statistically significant decrease in ABCB1 protein expression was observed in COLO 320 cells at all exposure times: at 24 h (*p* = 0.025), 48 h (*p* = 0.009), and 72 h (*p* = 0.003), suggesting the compound’s potential role in efflux inhibition. Additionally, a significant reduction in ABCC1 expression was observed in COLO 320 cells after 48 h of treatment (*p* = 0.047). In contrast, ABCB1 expression was undetectable in COLO 205 cells, indicating differential ABC transporter expression across the tested cell lines.

Furthermore, a significant decrease in GAL1 expression was observed in COLO 205 cells at 24 h post-treatment (*p* = 0.009), suggesting additional cellular targets of the 1C acridine-based chalcone compound.

#### 2.2.2. Immunofluorescence Analysis of ABCB1

**Figure 3:**
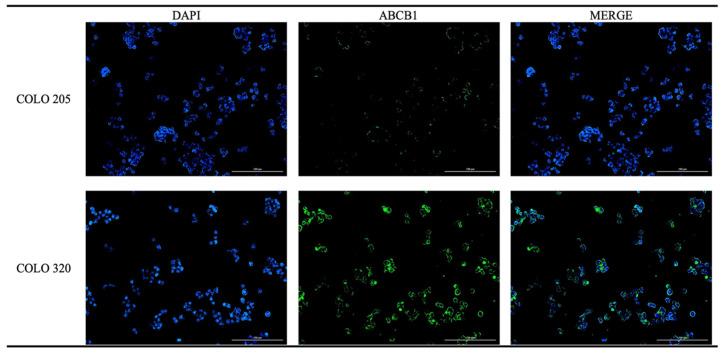
Immunofluorescence analysis of the presence of ABCB1 in COLO 205 and COLO 320 cells. Green indicates the presence of the transporter; blue indicates the nuclei. Representative images. Magnification 10×. Scale bar = 200 μm.

To verify the differential expression of the ABCB1 transporter observed in the Western blot analysis of the COLO 205 and COLO 320 cell lines, we conducted an immunofluorescence assay. The results demonstrated a distinct difference in expression levels of ABCB1 between the COLO 205 and COLO 320 cell lines (Figure 3).

#### 2.2.3. Chalcone 1C and ABCB1 Gene Expression

The effect of compound 1C (5 µmol/L) on the gene expression of ABC transporters was analyzed in the COLO 320 cell line using a quantitative reverse transcription polymerase chain reaction (qRT-PCR) assay. The results showed a significant decrease in *ABCB1* gene expression in the experimental group compared to the control, particularly within the first 24 h. The fold change was 0.44 (Table 2), which indicates a 2.27-fold downregulation of *ABCB1* gene expression after treatment. Although a downward trend in *ABCB1* expression persisted at 48 h post-treatment, the reduction did not reach statistical significance.

The finding suggests that the reduction in ABCB1 protein expression observed in the Western blot analysis may be partly attributed to the substance’s impact on gene expression.

#### 2.2.4. 1C and ATPase Activity Assay

**Figure 4:**
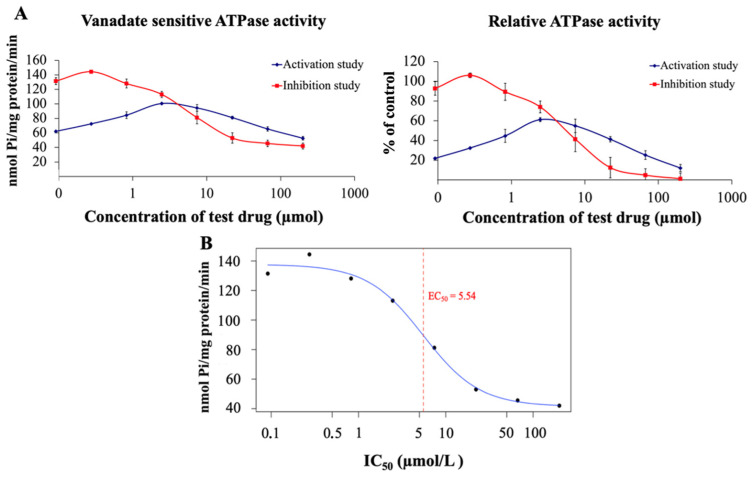
(**A**) Result of the ATPase assay displaying the dose–response curve of the 1C chalcone derivative on ABCB1-linked ATPase. Activity of ATPase is measured through the concentration of released inorganic phosphate (Pi). (**B**) The half-maximal effective concentration (EC_50_) for the 1C derivative was determined to be 5.53 ± 0.97 µmol/L.

The adenosine triphosphatase (ATPase) assay was used to measure the type of interaction of the tested chalcone derivative with the human ABCB1 transporter. The inhibition test of the 1C chalcone derivative, which was conducted with verapamil as an activator of the ABCB1 transporter, yielded a characteristic inhibition curve (Figure 4A). Furthermore, the half-maximal effective concentration (EC_50_) for ABCB1 inhibition for the 1C derivative was determined to be 5.53 ± 0.97 µmol/L (Figure 4B). These results indicate that 1C could act as an efficient concentration-dependent inhibitor of ABCB1 transport activity (an intermediate inhibitor) or as a slowly transported substrate. The activation test showed a biphasic curve that first increases and then decreases (Figure 4A). This biphasic phenomenon suggests that the compound exhibits dual effects on ABCB1 ATPase activity: an initial activation of ATPase activity at low drug concentrations followed by the inhibitory effect of 1C at high concentrations. The findings point to the compound potentially functioning as a competitive inhibitor, partial substrate, or allosteric modulator of the ABCB1 transporter.

#### 2.2.5. Docking Analysis of 1C

**Figure 5:**
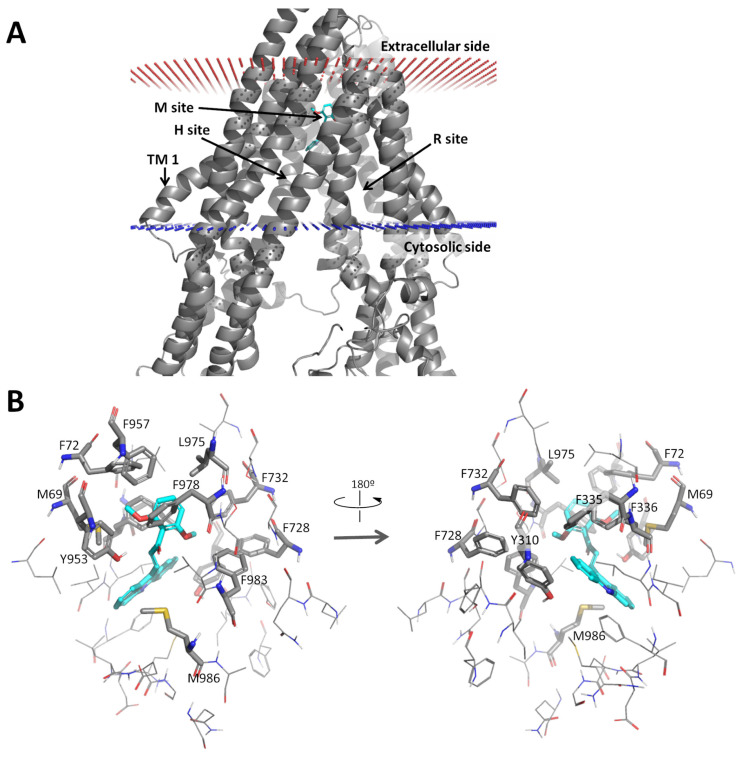
Docking results of compound 1C and the human ABCB1 protein in different visualizations: (**A**) transmembrane region of ABCB1 and (**B**) M binding site residues interacting with 1C. The protein is represented by gray cartoons or sticks; the compound is represented by colored sticks.

Next, a docking study of the 1C compound was performed on the ABCB1 efflux pump. The binding modes of the 1C acridine-based chalcone derivative was in the modulator site (M-site) of ABCB1, within the transmembrane region (Figure 5A).

The compound formed mainly aromatic and hydrophobic interactions with M69, F72, Y310, F335, F336, F728, F732, Y953, F978, F983, L975, and M986 (Figure 5B). According to the experimental data, the acridine moiety is well accommodated in the hydrophobic subpocket defined by M69, F72, F336, F732, Y953, F957, L975, and F978. The docking calculations predicted a relatively strong affinity of compound 1C with the human ABCB1 protein: ΔG_bind_ of −8.5 kcal/mol (via AutoDock Vina) and a dissociation constant of 40.1 nM (via NNScore).

### 2.3. Effect of 1C on ABCC1 and ABCG2

The effects of chalcone derivative 1C (5 µmol/L) on the ABCC1 and ABCG2 transporters were examined at both the protein and gene expression levels in COLO cell lines.

#### 2.3.1. Chalcone 1C and the Protein Expression of ABCC1 and ABCG2

Western blot analysis showed a significant reduction in ABCC1 protein levels in COLO 320 cells after 48 h of incubation with compound 1C, whereas no notable changes were observed in COLO 205 cells (Figure 2). Considering its inhibitory effect on the ABCB1 protein, these findings suggest that 1C may have a dual impact on two ABC transporters. Additionally, ABCG2 protein levels remained unchanged in both tested cell lines.

Overall, the results indicate that acridine-based chalcone 1C may modulate transporter protein expression in a manner dependent on both time and cell type.

#### 2.3.2. Chalcone 1C and the Gene Expression of ABCC1 and ABCG2

qRT-PRC analysis of COLO 320 cells revealed that the expression levels of the *ABCC1* and *ABCG2* genes remained unchanged after 24 and 48 h of treatment with chalcone 1C in COLO cells (Table 2).

The lack of significant changes in *ABCC1* and *ABCG2* gene expression suggests that the treatment’s effects may be specific to the *ABCB1* gene. Furthermore, the absence of an inhibitory effect of 1C on *ABCC1* expression, despite a decrease in the corresponding protein levels after 48 h of incubation, suggests the involvement of an alternative regulatory pathway rather than direct gene expression modulation.

#### 2.3.3. Effect of 1C on *Galectin*-1 Expression

Western blot analysis was finally conducted to evaluate GAL1 levels in COLO cell lines following incubation with compound 1C at a concentration of 5 µmol/L for 24, 48, and 72 h. We observed a significant decrease (*p* < 0.01) in the expression of GAL1 in COLO 205 cells after 24 h of exposure to the substance studied (Figure 2). Notably, GAL1 was not detectable in the COLO 320 cell line (Figure 2).

The results indicate that chalcone derivative 1C can modulate the expression of GAL1 in a time-dependent manner, depending on the cell phenotype.

### 2.4. Functional Efflux Assay in COLO 205 and COLO 320

**Figure 6:**
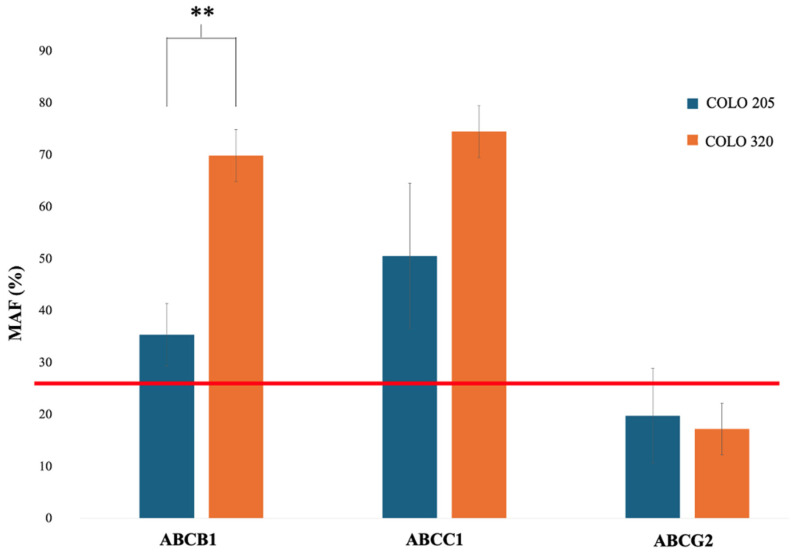
Multidrug resistance activity factors (MAFs) of ABC transporters. The red line represents the threshold, below which the transporter is considered non-functional. Results were obtained from at least three independent experiments. The graph was constructed using the geometric means of MAF. Significance level ** *p* < 0.01.

Flow cytometry was used to evaluate the presence and activity of ABC transporters in COLO 205 and COLO 320 cells by measuring fluorescence intensity after incubation with a fluorescent substrate, with or without specific ABC transporter inhibitors. Among all transporters, only ABCB1 and ABCC1/2 exhibited the calculated multidrug resistance activity factor (MAF) values higher than the manufacturer’s activity threshold (Figure 6 and Table S6), which is set at 25. Furthermore, the results showed that the MAF value for ABCB1 was significantly higher in the COLO 320 cell line (*p* = 0.005) than in COLO 205 cells. No significant change was observed in the activity of ABCC1/2 (*p* = 0.105) and ABCG2 (*p* = 0.680) between the tested cell lines (Figure 6).

**Figure 7:**
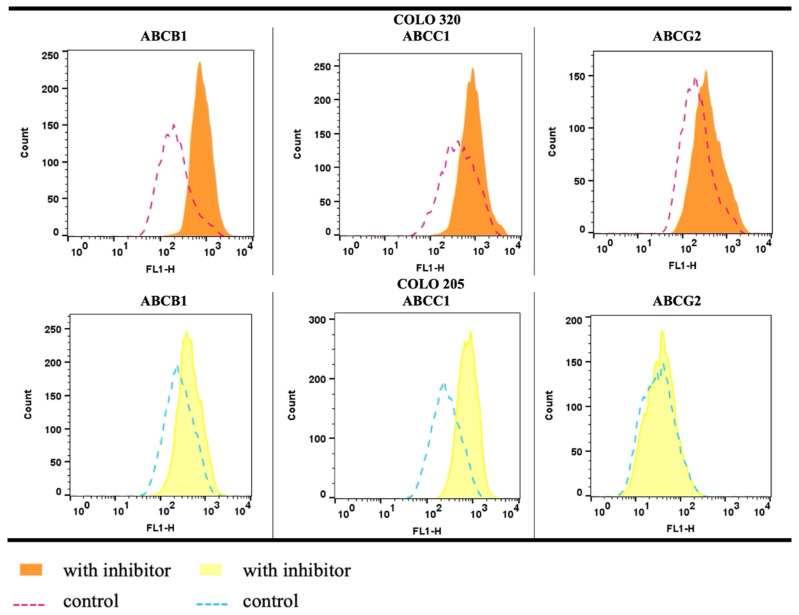
Histograms from the EFLUXX-ID^®^ Green multidrug resistance assay for ABC transporter activity. The histograms illustrate changes in the retention of the fluorescent substrate (Efflux-ID Green dye). The non-tinted histograms represent the fluorescence of untreated COLO 320 and COLO 205 cells, while the tinted histograms correspond to cells treated with the specific ABC transporter inhibitors: verapamil (for ABCB1), MK-571 (for ABCC1/2), and novobiocin (for ABCG2). Representative histograms of 3 experiments per group.

Flow cytometric histograms of cells incubated with Efflux-ID Green dye revealed a pronounced rightward shift in the presence of verapamil and MK-571, whereas novobiocin caused only a slight shift (Figure 7).

The findings indicate that both cell lines express all three transporters, with efflux activity primarily associated with ABCC1/2 and ABCB1 pumps. Notably, even the doxorubicin-sensitive COLO 205 cell line exhibited ABCB1 activity, albeit at a lower level than the resistant COLO 320 cells.

## 3. Discussion

Chalcones, including acridine-based derivatives, represent a noteworthy class of compounds with strong anticancer potential [27,29,34,39,40,53,54,55]. Recently, we were the first to demonstrate that the novel acridine-based chalcone analog 1C inhibits the proliferation of human colon adenocarcinoma cells overexpressing the ABCB1 transporter and effectively blocks the efflux of its substrate [41]. Moreover, our research demonstrated that 1C was a more potent inhibitor of ABCB1 than verapamil and exhibited synergy with doxorubicin in the COLO 320 cell line. In this study, we primarily focused on evaluating the effect of 1C on the protein and gene expression of ABCB1 in colon cancer cells, as well as on studying its possible molecular interaction potential with this transporter. At the same time, the effect of 1C on the protein and gene expression of two other important efflux transporters (ABCC1 and ABCG2) was also investigated. Another goal was to assess the effect of the test compound on galectin-1 (GAL1) protein expression.

Initially, to validate the ability of the 1C compound to target cancer cells while sparing healthy non-cancerous cells, cell viability assays were conducted on two previously untested healthy cell lines (BJ-5ta and FHC). The inhibitory effects of 1C on the cell viability of non-cancerous cell lines were significantly reduced in comparison with the effects on cancer cells (COLO 205, COLO 320, and HT-29), which highlight the compound’s selective cytotoxicity and promising safety. Such a preferential action of chalcones, including compound 1C, has also been reported in other works [40,41,56,57,58,59,60,61,62,63,64].

In subsequent experiments, we assessed the effects of chalcone 1C on the protein and gene expression of ABCB1 in COLO 320 cells. Notably, no detectable ABCB1 protein expression was observed in the COLO 205 cell line, which was consistent with previous studies [65]. An immunofluorescence assay further confirmed the low expression of the ABCB1 protein in these cells. Conversely, data from the Human Protein Atlas support the presence of ABCB1 protein expression in COLO 320 cells [66]. Following incubation with chalcone 1C, COLO 320 cells exhibited a statistically significant reduction in ABCB1 protein level across all exposure durations. This suggests that the decreased substrate efflux through this transporter [41] may be linked to a reduction in its protein levels. However, ABCB1 gene expression was significantly downregulated only after 24 h of incubation with 1C. Other chalcones exhibited diverse effects on ABCB1 expressions. For example, licochalcone A and flavokawain A reduced ABCB1 protein levels in BT-20 breast cancer cells and A549/T lung cancer cells, respectively [67,68]. In contrast, ABCB1 expression remained unaffected by trans-chalcone in BT-20 [67] and by CYB-2 and B20 in cervical cancer cells [69,70]. Notably, changes in ABCB1 protein expression do not always correlate with gene expression or transporter activity [68], underscoring the complexity of ABCB1’s regulatory mechanisms.

The effect of chalcone 1C on the ATPase activity of the ABCB1 transporter was evaluated using a commercial ATPase activity assay. This test, which measures the vanadate-sensitive portion of total ATPase activity, is employed to assess the interaction of test compounds with human ABCB1 in purified membrane vesicles that specifically overexpress this ABC transporter. It is widely recognized that ABCB1’s transport function relies on ATPase activity, as ATP binding and hydrolysis induce conformational changes necessary for substrate translocation [71,72]. Therefore, changes in ATPase activity following incubation with a compound can indicate whether it acts as a substrate or inhibitor of ABCB1 [73]. Based on this principle, the impact of chalcone 1C on ABCB1 ATPase activity was examined. In the inhibition test, chalcone 1C induced a concentration-dependent decrease in verapamil-stimulated ATPase activity. Meanwhile, in the activation assay, a biphasic response was observed, with an initial increase in ATPase activity followed by a decline. According to the literature, ABCB1 modulators are low-molecular-weight compounds (typically under 450 g/mol) that can either stimulate or inhibit transporter ATPase activity depending on their concentration [74]. Given that chalcone 1C has a molecular weight of 369.413 g/mol, these findings suggest that it functions as an ABCB1 modulator rather than a direct inhibitor. In comparison, xanthohumol has been reported to enhance ABCB1 ATPase activity [75].

To gain a deeper insight into the structural interactions between acridine-based chalcone 1C and the ABCB1 transporter, an in silico docking study was performed. Molecular docking has become a valuable computational tool in drug discovery [76], particularly for predicting drug interactions with ABC transporters like ABCB1 [19,24,77,78]. Its growing popularity stems from its ability to save time, reduce labor, and lower costs in drug development [79]. Recently, members of our research team published a comprehensive study in which they (1) modeled the full-length human ABCB1 transporter, refining it using molecular dynamics; (2) developed a docking protocol based on known natural lignans, and (3) virtually screened nearly 90 natural flavonoids using this protocol, some of which were proved experimentally to be effective in increasing the accumulation of anticancer drug doxorubicin inside promyelocytic leukemia HL60/MDR cells [80]. Building on this work, they conducted a docking study of the acridine-based chalcone analog 1C within ABCB1 to assess its binding modes and affinity, which is reported herein. The results revealed that compound 1C mainly binds to the M site of the human ABCB1 transporter, displaying a relatively strong affinity for the protein. ABCB1 contains multiple binding sites, with the M site located in the transmembrane region, acting as a crucial modulator-binding site that influences conformational changes [81]. Additionally, chalcone 1C interacts with the Y310 and Y953 residues of ABCB1, which serve as active sites involved in transporter inhibitor binding [19]. Currently, ABCB1 inhibitors are categorized into several types based on their binding sites: (1) competitive inhibitors bind directly to drug-binding sites; (2) non-competitive inhibitors modify ABCB1 conformations through allosteric residues, which is known as allosteric inhibition; (3) ATP-binding inhibitors prevent ATP binding, also leading to noncompetitive inhibition; and (4) membrane-interacting compounds disrupt the lipid bilayer environment [43,82]. Another inhibition mechanism involves the downregulation of ABCB1 expression by targeting its upstream regulators, a process that is not directly linked to the transporter itself [83]. Based on our computational analysis of drug–protein interactions and findings from the ATPase assay, we propose that chalcone 1C may inhibit efflux primarily as an allosteric modulator. Recent studies have also emphasized that substances binding on the TMD of ABCB1 can trigger conformational changes in the NBD, potentially affecting ATP hydrolysis [84]. Further structural analysis indicated that the allosteric interaction between the TMDs and NBDs is mainly mediated by two intracellular coupling helices. Interestingly, flavonoids and flavanone derivatives, which share structural similarities with chalcones, have also been identified as potential conformational modulators of the ABCB1 efflux pump [85,86].

In addition to ABCB1, efflux transporters ABCG2 and ABCC1 are also well known for their role in cancer chemoresistance [87]. Therefore, the effects of chalcone 1C on the protein and gene expression of these two transporters were investigated. The compound was found to reduce ABCC1 protein levels in COLO 320 cells after 48 h of incubation, with no other significant changes observed. Given that chalcone 1C also inhibited ABCB1, this finding suggests the possibility of a dual mechanism of action. The dual effect of 1C on the ABCB1 and ABCC1 transporters might be explained by the recent classification of ABC transporters, which groups them based on TMD folding. According to this classification, ABCB1 and ABCC1 belong to the same category, type IV, while ABCG2 is classified as type V [88]. As observed with ABCB1, the inhibition of ABCC1 and ABCG2 proteins appears to depend on both the specific chalcone and on the cell line tested. Xanthohumol reduced ABCG2 protein levels in doxorubicin-resistant breast cancer cells [89], while CYP-2B had no effect in NCI-H460/MX20 lung carcinoma cells [69]. Isoliquiritigenin downregulated ABCC1 in Caco-2 colon cancer cells but not in leukemic cells, whereas liquiritigenin increased ABCC1 in both [90]. In breast cancer cells, trans-chalcone and licochalcone A decreased ABCC1 expression in MCF-7 cells and downregulated ABCG2 (but not ABCC1) in BT-20 cells [67].

The next objective was to evaluate the impact of 1C on galectin-1 (GAL1) protein expression. Galectins are key regulators of cancer progression, influencing processes such as cell growth, migration, invasion, angiogenesis, and therapy resistance [48,91,92,93,94,95,96,97]. Elevated GAL1 levels have been associated with lymph node metastasis, cancer development, and a poor prognosis, particularly in colorectal cancer [98,99,100]. Several studies have also emphasized the role of galectins in regulating the expression and activity of ABC transporters [47,49,50,101]. Given their significance in carcinogenesis, galectins, primarily GAL1 and GAL3, are emerging as promising therapeutic targets [92,102,103,104]. Galectin inhibitors have the potential to enhance anticancer treatments when combined with chemotherapy [105], radiation [106], immunotherapy, and antiangiogenic therapy [107]. Previously, our preliminary research (unpublished data) did not establish any effect of chalcone 1C on GAL3 in COLO 205 and COLO 320 cells, prompting us to shift our focus to GAL1. In the present study, we observed a significant difference in GAL1 protein expression across COLO cell lines. In COLO 320 cells, which overexpress ABCB1, GAL1 was undetectable, whereas it was present in the parental COLO 205 cells. This observation is consistent with data from the Protein Atlas, which also confirmed the absence of the GAL1 protein in COLO 320 cells [108]. Furthermore, previous research validated GAL1 mRNA expression in COLO 205 cells [109]. The impact of ABCB1 expression on GAL1 levels in colorectal cancer cells is not yet understood. Future experimental studies, such as knocking down ABCB1 in the COLO 320 cell line or introducing ABCB1 into COLO 205 cells, could help to clarify whether changes in ABCB1 expression influence galectin levels. Interestingly, previous studies comparing ABCB1-positive and ABCB1-negative cell lines found that ABCB1 overexpression downregulated another galectin, galectin-9 [110]. These researchers also identified multiple isoforms of the studied galectin, revealing distinct protein expression profiles between ABCB1-positive and ABCB1-negative cells. Furthermore, our findings indicate that chalcone 1C significantly reduced GAL1 protein expression in COLO 205 cells after 24 h of incubation, suggesting an additional potential mechanism of its anticancer activity in certain cell lines. Based on the results, it is necessary to clarify the effects of compound 1C on the levels of GAL1 even at shorter incubation times in the future. Although the effects of chalcones on GAL1 remain largely unexplored, some studies suggest that chalcone-derived compounds may inhibit this protein [51]. However, the mechanism underlying the reduction in galectin levels by these substances has not been studied. Notably, chalcone-based compounds have been shown to inhibit another galectin, GAL3 [52]. Since elevated GAL1 levels are more frequently linked to other gastrointestinal tumors and malignancies outside of colorectal cancer [95,97,102], further studies are necessary to evaluate the effect of compound 1C on GAL1 expression across additional cell lines. However, recent research on pancreatic ductal carcinoma reported that conversely higher ductal cellular expression of GAL1 correlated with smaller tumor sizes and improved patient survival [111]. The study also highlighted the variable expression of galectins in different cell types, emphasizing the need for cancer type-specific investigation, including assessments of circulating galectin levels. As compound 1C significantly inhibited the expression of ABCB1 and ABCC1 only in the cell line without GAL1 expression in our experiments, the correlation between changes in the protein levels of transporters and GAL1 could not be determined.

Finally, we assessed efflux transporter activity in the COLO 205 and COLO 320 cell lines using a commercial kit. While all transporters were present, only ABCB1 and ABCC1/2 showed significant activity, with the higher multidrug activity factor (MAF values) observed in COLO 320. ABCC1/2 had the highest activity, while ABCG2 showed minimal involvement in drug resistance. These findings align with previous studies and the kit manufacturer’s data in other colorectal cancer cell lines [112,113]. However, in HCT-15 cells, the MAF was above 25% solely for the ABCB1 transporter. The LoVo cell line, which is resistant to doxorubicin, exhibited activity for all three transporters [114]. These results suggest that the presence and activity of efflux transporters in various colon carcinoma-derived cell lines are cell type-dependent and can be further activated by the administered drugs. Interestingly, we detected ABCB1 activity in COLO 205 despite low or undetectable protein levels. This finding underscores a discrepancy between protein expression and functional activity, which is consistent with observations reported in other studies [113,114,115]. Several factors may account for this phenomenon, including the dependence of protein expression on the cell cycle phase, the limited sensitivity or specificity of antibodies, variations in antigen conformation depending on the applied methods, differences in protein density across specific cell lines, the restricted selectivity of substrates and inhibitors in functional assays, the potential induction of efflux transporters by chemical agents, or posttranslational modifications of ABC proteins [114,116]. As a result, an evaluation of both protein expression and transporter activity is strongly recommended for a more comprehensive analysis [114].

### Limitations and Future Directions

The main limitation of our study is the performance of experiments on classical 2D in vitro models, which may not fully reflect the complexity of tumor behavior in vivo (e.g., a lack of the tumor microenvironment or the absence of the immune system). The quantitative structure–activity relationship analysis (QSAR), 3D tumor spheroid models, organoids, co-culture systems, or organ-on-a-chip method could be advanced alternatives before performing in vivo experiments [117]. Furthermore, this study focuses only on individual transporters in selected cells and does not evaluate potential off-target effects and a broader pharmacokinetic profile, which are essential for clinical applications. Therefore, there is a need to determine whether chalcones, including acridine-based analogs, are equally effective and selective in humans as in laboratories. Comprehensive toxicity profiling and pharmacokinetic studies will be essential to assess the clinical feasibility of chalcone 1C as a therapeutic agent. Overall, potential future research should aim to validate the observed in vitro effects of chalcone 1C in relevant in vivo models of colorectal cancer to better reflect physiological conditions.

Given the fact that several anticancer drugs are substrates of ABC transporters [7] and are often administered in combination treatment, it is necessary to thoroughly investigate the interaction potential of chalcone 1C. Although we have previously noted the synergism of the substance with doxorubicin [41], combinatorial strategies involving the chalcone and anticancer agents should be explored to evaluate synergistic effects and potential improvements in treatment efficacy.

Due to the poor solubility and low bioavailability of chalcone compounds [118], there is also a need to develop suitable pharmaceutical formulations.

Since some effects of 1C were observed as early as 24 h, future studies should explore chalcones at shorter incubation times. Moreover, the effects of 1C, as well as other acridine-based chalcone analogs, on ABC transporters in different cell lines should be assessed.

Next, an investigation into the molecular mechanisms underlying the interactions between chalcone 1C, ABC transporters, and GAL1 is warranted. It remains to be clarified whether the effects of 1C on ABC transporters and galectin-1 are mediated through direct transcriptional repression, post-transcriptional regulation (e.g., via microRNAs), epigenetic modifications, altered translational control, alternative splicing, enhanced protein degradation, or the modulation of upstream regulatory signaling pathways. Additionally, the relationship between the ABCB1 transporter and GAL1 requires further elucidation. Expanding the analysis to include additional ABC transporters and galectin family members may reveal broader implications for overcoming chemoresistance.

Moreover, comprehensive toxicity profiling and pharmacokinetic studies will be essential to assess the clinical feasibility of chalcone 1C as a therapeutic agent.

Recently, the existence of multiple isoforms of ABC transporters has been revealed [12]. Therefore, another drawback is a lack of information about the exact isoform of the transport proteins studied in our research. The lack of antibodies to individual isoforms or the cross-reactivity of antibodies available on the market may also be another obstacle.

Unfortunately, efflux transporters can often contribute to drug resistance simultaneously. Thus, chalcones with dual or multiple modulatory actions could provide an advantage [36,69]. As shown in the recent literature, new effects not related to anti-efflux activity have been identified for ABC inhibitors [22], which may require further analysis to elucidate the molecular mechanisms of action.

Next, another limitation of studying the activity of transport proteins is the overlapping of their substrate specificity. A great challenge therefore represents the discovery or synthesis of new fluorescent substances useful for scientific purposes, which would be a specific substrate of only one ABC transporter. Promising molecules appear to be, e.g., the TO-PRO™-1 and TO-PRO™-3 dyes, whose intracellular accumulation was shown to be exclusively influenced by the ABCB1 transporter, with no dependence on the ABCC1 and ABCG2 transporters [119]. Additionally, cancer treatment with traditional cytostatic agents is often associated with an increased risk of serious infections due to neutropenia, which requires the administration of antibiotics. Drug efflux is well known as one of the possible mechanisms of resistance to these drugs. It is therefore appropriate to investigate chalcones also in the context of their effect on inhibiting efflux in bacteria and other microbial organisms. Such effects have already been recorded for some chalcone molecules [120,121,122,123]. The dual modulation of drug efflux in cancer and bacterial cells could be an advantage.

Finally, in silico approaches and artificial intelligence (e.g., machine learning models) should be employed to optimize compound design and guide the development of more potent and safe analogs [122,124,125].

## 4. Materials and Methods

### 4.1. Test Compound

The chalcone analog (2E)-3-(acridin-9-yl)-1-(2,6-dimethoxyphenyl)prop-2-en-1-one (1C, Figure S1) was synthesized at the Institute of Chemistry, Faculty of Science, P. J. Šafárik University in Košice, Slovakia. The compound’s structure was confirmed through ^1^H and ^13^C NMR, IR spectroscopy, and mass spectrometry. For experimental use, it was dissolved in DMSO (Sigma-Aldrich, St. Louis, MO, USA) to achieve a final, non-toxic concentration of 0.05% The stock solutions were diluted in the culture medium immediately before the experiments.

### 4.2. Cell Culture

The study utilized several cell lines, including COLO 205 (CCL-222), COLO 320 (CCL-220), and HT-29 (HTB-38) as cancer cells and BJ-5ta (CRL-4001) and FHC (CRL-1831) as non-cancerous cells. The COLO 205 cell line (a human colorectal adenocarcinoma with an epithelial morphology) and the COLO 320 cell line (a multidrug resistant variant overexpressing ABCB1) were kindly provided by associate professor Gabriella Spengler (Department of Medical Microbiology, Albert Szent-Györgyi Medical School, University of Szeged, Hungary). The HT-29 cell line (human colorectal adenocarcinoma with epithelial morphology), the BJ-5ta cell line (fibroblast cells isolated from the foreskin), and FHC cells (healthy fetal human colon cells with epithelial morphology) were obtained from the American Type Culture Collection (ATCC, Manassas, VA, USA). The COLO 205, COLO 320, and BJ-5ta cells were cultured in the Roswell Park Memorial Institute (RPMI) 1640 medium (Biosera, Kansas City, MO, USA), while the HT-29 and FHC cell lines were grown in DMEM-F12 (Sigma-Aldrich, St. Louis, MO, USA). All media were supplemented with HyClone™ antibiotic/antimycotic solution (GE Healthcare, Piscataway, NJ, USA) and 10% fetal bovine serum (FBS) (Invitrogen, Carlsbad, CA, USA). Additionally, the medium for FHC cells required the addition of 0.005 mg/mL insulin, 0.005 mg/mL transferrin, 100 ng/mL hydrocortisone, 20 ng/mL human recombinant EGF, and 10 ng/mL cholera toxin (all compounds obtained from Sigma-Aldrich, St. Louis, MO, USA). Cells were maintained under standard conditions at 37 °C in a 5% CO_2_ atmosphere.

### 4.3. Molecular Docking

The three-dimensional model of human ABCB1 was previously prepared through molecular threading (I-TASSER [126]) and refined via a 500 ns molecular dynamics simulation. After clustering the snapshots and docking the known ABCB1 inhibitors, the best representative structure was selected. This structure (cluster5 in the original work) was used here without further modifications [80]. The three-dimensional structures of the ligands to be docked were obtained from the PubChem database [127]. The semi-empirical AM1-BCC function [128,129] was employed to calculate the partial charges of the ligands using the antechamber module of AmberTools 16 [130]. The input files of the ligands and receptors, in MOL2 and PDB formats, respectively, were converted to the AutoDock Vina-compatible format PDBQT using MGLTools software, version 1.5.4 [131], thereby maintaining the previously calculated atomic charges of the ligand; the non-polar hydrogens of the ligands were kept in the structures. The binding site of the human ABCB1, as identified by I-TASSER (residues 69, 72, 336, 40, 343, 725, 728, 732, 953, 975, 978, 979, 983, and 986), was used to define the region of interest for the molecular docking performed using AutoDock Vina 1.1.2 [132]. This region was represented by a box of 40 × 40 × 40 Å centered at the coordinates of the center of mass of the Cα atoms of the binding site residues. The exhaustiveness parameter was increased to 100 to improve the conformational search. The maximum number of binding modes also increased to 20. The docking results were visualized using PyMOL 2.3.2. [133]. The results were analyzed based on the binding energy (ΔG_bind_) of the best predicted conformations calculated using AutoDock Vina, which were re-scored using NNScore to predict the respective dissociation constants (*K*_d_).

### 4.4. Viability Assays

The antiproliferative activity and IC_50_ values of the test compound were assessed using the methylthiazoltetrazolium (MTT) colorimetric assay (3-(4,5-dimethylthiazol-2-yl)-2,5-diphenyltetrazolium bromide, Sigma-Aldrich, St. Louis, MO, USA). The COLO 205 and COLO 320 cell lines were seeded in 96-well plates at a density of 4800 cells/cm^2^ and incubated for 24 h to allow for cell attachment. The chalcone analog 1C was tested at concentrations of 3.125, 6.25, 12.5, and 25 µmol/L for both cell lines, while for the BJ-5ta cell line, 1C was tested at concentrations of 1, 10, 25, 50, and 100 µmol/L. The incubation periods were 24, 48, and 72 h for COLO 205 and COLO 320, while the BJ-5ta cell line was treated for 72 h. After each incubation, 10 µL of the MTT solution (5 mg/mL) was added to each well, followed by 4 h of incubation at 37 °C to allow for formazan crystal formation. To dissolve the crystals, 100 µL of 10% sodium dodecyl sulfate (SDS, Sigma-Aldrich, St. Louis, MO, USA) was added, and plates were further incubated for 24 h at 37 °C.

Additionally, the sulforhodamine B (SRB, Sigma-Aldrich, Poznan, Poland) assay was used to evaluate cell viability in the HT-29 and FHC cell lines at the Department of Biophysics and Neurobiology, Wroclaw Medical University, during an Erasmus+ program internship (version KA131). HT-29 cells were seeded at 38,400 cells/cm^2^ and treated with 1C at concentrations of 1, 5, 10, 25, and 75 µmol/L, while FHC cells were seeded at 57,600 cells/cm^2^ and treated with 1C at 5, 25, 50, 100, and 150 µmol/L. After 48 h of exposure, cells were fixed with 50 µL of 50% ice-cold trichloroacetic acid (TCA, Sigma-Aldrich, St. Louis, MO, USA) per well and incubated at 4 °C for 1 h. Plates were then rinsed thoroughly, air-dried, and stained with 100 µL of the 0.02% SRB solution (prepared in 1% acetic acid, Sigma-Aldrich, St. Louis, MO, USA) for 1 h at room temperature. Excess dye was removed by washing with 1% acetic acid until the solution ran clear, and plates were dried again. The dye was solubilized with 150 µL of the 10 mmol/L TRIS-base solution (Sigma-Aldrich, St. Louis, MO, USA) (pH 10.4–10.5), and absorbance was measured at 540 nm for MTT or 492 nm for SRB using a Cytation™ 3 Cell Imaging Multi-Mode Reader (Biotek, Winooski, VT, USA). IC_50_ values were calculated using nonlinear regression from three independent experiments.

The selectivity index (SI) was calculated to assess the preferential cytotoxicity of the test compound. SI was defined as the ratio of the IC_50_ values for non-cancerous cell lines to the corresponding IC_50_ values for cancer cell lines.

### 4.5. Immunofluorescence

Cells were cultured and fixed using a methanol/acetone solution (1:1) for 15 min at 4 °C. Slides were air-dried and hydrated by washing with 1% Triton X-100 (Sigma-Aldrich, St. Louis, MO, USA) for 10 min; the procedure was repeated three times. Goat serum (Biosera, Cholet, France) was applied to prevent nonspecific staining, followed by three washes with 1% Triton X-100. Primary antibodies, which were prepared according to the manufacturer’s recommended dilutions (Table 3), were added and incubated overnight at 4 °C. Slides were washed, and a secondary antibody was applied for 60 min at room temperature. After washing three times, Hoechst dye 33342 (Merck, Burlington, MA, USA) was added for 20 min before imaging on the Cytation™ 3 Reader (Biotek, Winooski, VT, USA). This experiment was conducted in duplicate to ensure the consistency and reliability of the results.

### 4.6. ATPase Activity Analysis

The MDR1 PREDISEASY ATPase Assay Kit (Solvo Biotechnology, Budapest, Hungary) was used to assess the ATPase activity of the ABCB1 transporter in response to the test compound. The assay was performed according to the manufacturer’s instructions and included both activation and inhibition tests.

For the activation assay, MDR1-Sf9 membranes containing recombinant human ABCB1 were incubated with the assay buffer and increasing concentrations of the test compound (0.09, 0.27, 0.82, 2.47, 7.41, 22.22, 66.67, and 200 µM) to evaluate its ability to stimulate ATP hydrolysis. The reaction was carried out at 37 °C, and ATP hydrolysis was initiated by adding ATP. After incubation, the reaction was stopped using a colorimetric detection reagent. ATPase activity was measured by quantifying the release of inorganic phosphate (Pi). The activity was determined as the difference in reactions conducted with and without 1.2 mM sodium orthovanadate (vanadate-sensitive ATPase activity). The concentration of inorganic phosphate was measured at 600 nm using a Cytation™ 3 Cell Imaging Multi-Mode Reader (Biotek, Winooski, VT, USA).

For the inhibition assay, the membranes were pre-incubated with verapamil, a known ABCB1 substrate, to stimulate ATPase activity. The test compound was then added to assess its inhibitory effects on ATP hydrolysis. The assay followed the same detection method, with Pi release serving as an indicator of ABCB1 inhibition.

The tests were conducted in three independent experiments, and a manufacturer-provided template was used for this calculation. ATPase inhibition data from this template were utilized to determine and visualize the EC_50_ using R version 4.4.1 (2024-06-14) software.

### 4.7. Functional Assessment of ABC Proteins

The Efflux-ID Green assay (ENZO Life Sciences, Lausen, Switzerland) was employed to assess drug efflux activity mediated by the ABCB1 (MDR1), ABCC1/2 (MRP1/2), and ABCG2 (BCRP) transporters. The assay was performed following the manufacturer’s protocol. Prior to initiating the assay, all cells were maintained in a complete growth medium devoid of Phenol Red (OptiMEM with GlutaMAX, Thermo Fisher Scientific, Waltham, MA, USA) to ensure optimal assay conditions. Cells were harvested using trypsinization and pre-incubated in phosphate-buffered saline (PBS, Thermo Fisher Scientific, Waltham, MA, USA) containing 2% FBS for 5 min at room temperature. The assay buffer contained one of the following: DMSO (vehicle control), 5 µmol/L verapamil (ABCB1 inhibitor), 10 µmol/L MK-751 (ABCC1/2 inhibitor), or 50 µmol/L novobiocin (ABCG2 inhibitor). The Efflux-ID Green dye was then added, and the cells were incubated for 30 min at 37 °C. After incubation, samples containing 10,000 cells each were analyzed using fluorescence-activated cell sorting (FACS) on a BD FACSCalibur flow cytometer (BD Biosciences, San Jose, CA, USA). Fluorescence emissions were measured at 514 nm following excitation at 490 nm. Data analysis was conducted using the FlowJo™ software (version 10.0, Tree Star, Inc., Ashland, OR, USA, RRID:SCR_008520). Geometric mean fluorescence intensity (MFI) values were utilized to calculate the multidrug resistance activity factor (MAF) for each transporter using the equation provided by the assay kit manufacturer:

$$MAF=100\times \frac{MAF\left(transporter\right)-MAF(control)}{MAF\left(transporter\right)}$$

Each experiment was performed at least three times to ensure reproducibility and reliability. The MAF values from all three experiments were averaged to determine the mean MAF value for each transporter. MAF values below 20 were considered indicative of multidrug resistance-negative specimens, while values above 25 were classified as multidrug resistance-positive specimens.

### 4.8. Quantitative Reverse Transcription PCR (qRT-PCR)

RNA was extracted from 3 × 10^6^ cells using the RNeasy Plus Mini Kit (Qiagen, Carlsbad, CA, USA) according to the manufacturer’s instructions, and RNA concentration and purity were measured with the NanoDrop 2000 spectrophotometer (Thermo Fisher Scientific, Waltham, MA, USA). A total of 2 μg of RNA was reverse transcribed into cDNA using the High-Capacity cDNA Reverse Transcription Kit (Applied Biosystems™, Foster City, CA, USA). Quantitative real-time PCR (qPCR) was performed using the TaqMan™ Fast Advanced Master Mix (Applied Biosystems™, Foster City, CA, USA) and specific TaqMan™ probes for the ABCB1 (Hs00184500_m1), ABCC1 (Hs001561483_m1), and ABCG2 (Hs01053790_m1) genes on the ABI 7500 Real-Time PCR System (Applied Biosystems™, Foster City, CA, USA). The housekeeping gene GUSB (Hs00939627_m1) (Applied Biosystems™, Foster City, CA, USA) served as the internal reference. Relative expression levels were calculated using the 2^−ΔΔCt^ method, with target gene Ct values normalized to GUSB and compared to untreated control samples. Each reaction was performed in triplicate, and no-template controls were included to confirm the absence of contamination.

### 4.9. Western Blot Analysis

Protein expression in COLO 320 and COLO 205 cells was evaluated using Western blotting. Cells were seeded in 10 cm Petri dishes (78.54 cm^2^) at a density of 1.5 × 10^6^ cells per dish. After 24 h of incubation, cells were treated with the 1C compound at a concentration of 5 µmol/L. Cells were collected at three time points: 24, 48, and 72 h. Protein lysates were prepared using the Laemmli lysis buffer containing 1 mol/L Tris/HCl (pH 6.8), glycerol, 20% SDS, deionized water, phosphatase, and protease inhibitors (Sigma-Aldrich, St. Louis, MO, USA). Lysates were sonicated, and protein concentrations were measured using the Pierce^®^ BCA Protein Assay Kit (Thermo Fisher Scientific, Waltham, MA, USA) at 570 nm with a Cytation™ 3 Reader. Proteins (30 µg per well) were loaded onto a 10% SDS-PAGE gel and electrophoresed at 115 V for three hours. Proteins were transferred to polyvinylidene fluoride (PVDF) membranes using the iBlot™ 2 Dry Blotting System (Invitrogen, Carlsbad, CA, USA). Membranes were washed in deionized water and blocked with 5% bovine serum albumin (BSA, SERVA, Heidelberg, Germany) in TBS-Tween (pH 7.4) for 1 h at room temperature. Primary antibodies (Table 4) diluted in 5% BSA or 5% non-fat dry milk (Cell Signaling Technology®, Danvers, MA, USA), as per the manufacturer’s instructions, were incubated with the membranes for 24 h at 4 °C. After rinsing (3 × 5 min in TBS-Tween), the membranes were incubated with HRP-conjugated secondary antibodies (Table 4) for 1 h at room temperature. Protein bands were visualized using an ECL substrate (Thermo Fisher Scientific, Waltham, MA, USA) and analyzed using the iBright™ FL1500 Imaging System (Thermo Fisher Scientific, Waltham, MA, USA). Protein expressions were normalized and evaluated against the loading control (β-actin). Western blot analysis was performed in three independent experiments.

### 4.10. Statistical Analysis

The data in this study are presented as the mean ± standard deviation (SD), calculated from at least three independent experimental replicates to ensure reliability. Statistical analyses were conducted using a *t*-test or one-way analysis of variance (ANOVA), depending on the data format, to assess differences among group means. A *p*-value of <0.05 was considered statistically significant, with significance levels denoted as follows: * *p* < 0.05, ** *p* < 0.01, *** *p* < 0.001, and **** *p* < 0.0001, in comparison to the DMSO-treated control group. Statistical analyses were performed using GraphPad Prism 8 Version 8.0.2 (GraphPad Software Inc., La Jolla, CA, USA) and R version 4.4.1 (2024-06-14), R Core Team. Data normality (ΔCt values) was assessed using the Shapiro–Wilk test, and differences between treated and untreated groups were evaluated using the non-parametric Kruskal–Wallis test, followed by Dunn–Bonferroni post hoc comparisons for multiple testing. Statistical significance was set at *p* < 0.05.

## 5. Conclusions

In summary, it is challenging to include chalcones with multiple anticancer effects, including inhibitory action on some ABC transporters, in future potential tumor treatment to achieve high antitumor efficacy, safety, and a reduction in drug resistance. This study identifies synthetic acridine-based chalcone analog 1C as an inhibitor of the ABCB1 transporter and suppressor of ABCC1 protein expression in colorectal cancer cells. Its dual targeting of functional ABC transporters, combined with selective antiproliferative effects on cancer cells, underscores 1C potential as a promising anticancer drug candidate. These findings highlight the need for further research to confirm our results and evaluate the clinical and interaction potential of chalcone 1C and its derivatives for cancer treatment, particularly in chemo-resistant cases. The significance of galectin 1 suppression in COLO 205 remains unclear, and the relationship between chalcone 1C, galectins, and various ABC transporters must be elucidated. Studying chalcones in relation to ABC transporters and galectins may contribute to a better understanding of the properties of these compounds.

## Figures and Tables

**Table 1:** The average half-maximal inhibitory concentrations values of compound 1C in non-cancer and cancer cells, as well as corresponding selectivity index values.

	MTT Assay (72 h)			SRB Assay (48 h)	
	BJ-5ta	COLO 205	COLO 320	FHC	HT-29
IC_50_ (µmol/L)	18.03 ± 1.23	5.34 ± 0.11	6.69 ± 0.7	>150	37.89 ± 0.63
*p*-value	Ref.	<0.0001	<0.0001	Ref.	<0.0001
Selectivity index	-	3.68 ± 0.16	2.71 ± 0.17	-	3.96 ± 0.07

BJ-5ta, human foreskin fibroblast cells; COLO 205, human colorectal adenocarcinoma cells; COLO 320, human colorectal adenocarcinoma cells overexpressing the ABCB1 transporter; FHC, human normal fetal colon epithelial cells; HT-29, human colorectal adenocarcinoma cells. The average half-maximal inhibitory concentrations (IC_50_) and selectivity index (IC_50_ of non-cancer cells/IC_50_ of cancer cells) values are reported as the mean ± SD from three independent experiments, determined using either the methylthiazoltetrazolium (MTT) colorimetric assay or the sulforhodamine B (SRB) assay.

**Table 2:** Comparative Ct (threshold cycle) method (−ΔΔCt) values and relative fold change in COLO 320 following 1C treatment.

Gene	Time (hours)	Mean	STDEV	*p*-Value	*p*-Value _adjusted_	RQ
*ABCB1*	24	1.17	0.05	0.002	0.013	0.44
	48	0.68	0.05	0.308	1	0.62
*ABCC1*	24	−0.21	0.12	0.308	1	1.16
	48	−0.72	0.32	0.213	1	1.65
*ABCG2*	24	0.62	0.09	1	1	0.65
	48	−0.26	0.02	1	1	1.20

STDEV, standard deviation; RQ, fold change.

**Table 3:** List of primary and secondary antibodies utilized in immunofluorescence visualization.

Antibody	MW (kDa)	Origin	Dilution	Catalog No.	Manufacturer
Anti-ABCB1	180	Rabbit	1:100	ab170904	Abcam, Cambridge, UK
Anti-Rabbit IgG Alexa Fluor™ 488	-	Goat	1:500	A11008	Thermo Fisher Scientific, Waltham, MA, USA

MW, molecular weight.

**Table 4:** List of primary and secondary antibodies used in Western blot analysis.

Antibody	MW (kDa)	Origin	Dilution	Catalog No.	Manufacturer
Anti-ABCB1	180	Rabbit	1:1000	ab170904	Abcam, Cambridge, UK
Anti-ABCC1	170–250	Rabbit	1:1000	ab233383	Abcam, Cambridge, UK
Anti-ABCG2	75	Rabbit	1:1000	ab207732	Abcam, Cambridge, UK
Anti-Galectin 1	15	Rabbit	1:2000	11858-1-AP	Proteintech, Manchester, UK
β-actin	45	Mouse	1:1000	3700S	Cell Signaling, Danvers, MA, USA
Anti-Rabbit HRP	-	Goat	1:1000	7074S	Cell Signaling, Danvers, MA, USA
Anti-Mouse HRP	-	Horse	1:1000	7076S	Cell Signaling, Danvers, MA, USA

MW, molecular weight.

## Data Availability

The data are contained within the article and Supplementary Material.

## Supplementary Materials

The following supporting information can be downloaded at: https://www.mdpi.com/article/10.3390/ijms26094138/s1.

## Abbreviations

The following abbreviations are used in this manuscript:

A549/T	Human paclitaxel resistant lung adenocarcinoma cell line
ABC	ATP-binding cassette
ABCB1	ATP-binding cassette subfamily b member 1 (P-glycoprotein)
ABCC1	ATP-binding cassette subfamily c member 1 (multidrug resistance-associated protein 1)
ABCG2	ATP-binding cassette subfamily g member 2 (breast cancer resistance protein)
ATPase	Adenosine triphosphatase
BJ-5ta	Human fibroblast cell line immortalized
BT-20	Human breast carcinoma cell line
Caco-2	Human colorectal adenocarcinoma cell line
COLO 205	Human colorectal adenocarcinoma cell line
COLO 320	Human multidrug resistant colorectal adenocarcinoma cell line
DMSO	Dimethyl sulfoxide
EC_50_	Half-maximal effective concentration
FHC	Fetal human colon cell line
GAL	Galectin
HCT-15	Human colorectal adenocarcinoma cell line
HT-29	Human colorectal adenocarcinoma cell line
IC_50_	Half-maximal inhibitory concentration
LoVo	Human colorectal adenocarcinoma cell line
MAF	Multidrug resistance activity factor
MCF-7	Michigan cancer foundation-7 breast cancer cell line
MRP1	Multidrug resistance-associated protein 1 (ABCC1)
MTT	Methylthiazoltetrazolium assay
NCI-H460/MX20	Human mitoxantrone resistant large cell lung carcinoma cell line
NBD	Nucleotide-binding domain
PBS	Phosphate-buffered saline
PVDF	Polyvinylidene fluoride
RPMI	Roswell Park memorial institute medium
SDS	Sodium dodecyl sulfate
SI	Selectivity index
SRB	Sulforhodamine B assay
TCA	Trichloroacetic acid
TMD	Transmembrane domain
qRT-PCR	Quantitative reverse transcription polymerase chain reaction
