# Supplementary material for: Acridine-Based Chalcone 1C and ABC Transporters
Source: Int J Mol Sci. 2025 Apr 27;26(9):4138. doi: 10.3390/ijms26094138 (PMC12071533; doi:10.3390/ijms26094138)

International Journal of
Molecular Sciences

MDPI

Acridine-Based Chalcone 1C and ABC Transporters

Ondrej Franko [1,†], Martina Čižmáriková [1,*,†], Martin Kello [1], Radka Michalková [1], Olga Wesołowska [2], Kamila Środa-Pomianek [2], Sérgio M. Marques [3,4], David Bednář [3,4], Viktória Háziková [1], Tomáš Ján Liška [5] and Viera Habalová [6]

[1] Department of Pharmacology, Faculty of Medicine, Pavol Jozef Šafárik University, 040 11 Košice, Slovakia
[2] Department of Biophysics and Neurobiology, Wroclaw Medical University, 50-369 Wrocław, Poland
[3] Loschmidt Laboratories, Department of Experimental Biology and RECETOX, Faculty of Science, Masaryk University, 625 00 Brno, Czech Republic
[4] International Clinical Research Center, St. Anne's University Hospital, 656 91 Brno, Czech Republic
[5] Institute of Chemistry, Faculty of Science, Pavol Jozef Šafárik University, 040 11 Košice, Slovakia
[6] Department of Medical Biology, Faculty of Medicine, Pavol Jozef Šafárik University, 040 11 Košice, Slovakia
* Correspondence: martina.cizmarikova@upjs.sk
† These authors contributed equally to this work.

Concentration 1C (μmol/L) BJ-5ta	Cell proliferation (%) BJ-5ta (72 hours)	*p*-Value
	119.01	
1	105.77	0.3616
	99.63	
	71.95	
10	71.21	0.0003
	68.32	
	36.98	
25	29.39	<0.0001
	29.39	
	32.63	
50	29.58	<0.0001
	18.48	
	30.94	
100	24.86	<0.0001
	19.12	

Table S1. Percentage of cell proliferation after 72-hour exposition of chalcone 1C in BJ-5ta, Human foreskin fibroblast cells as determined by the MTT assay. Compared to the DMSO treated control group (set to 100% proliferation).

Concentration 1C (μmol/L) COLO 205	Cell proliferation (%) COLO 205 (72 hours)	*p*-Value
3.125	69.63<br>59.34<br>71.59	<0.0001
6.25	43.43<br>45.96<br>39.94	<0.001
12.5	23.88<br>39.87<br>28.71	<0.0001
25	23.49<br>22.32<br>21.27	<0.0001

Table S2. Percentage of cell proliferation after 72-hour exposition of chalcone 1C in COLO 205, Human colorectal adenocarcinoma cells as determined by the MTT assay. Compared to the DMSO treated control group (set to 100% proliferation).

Concentration 1C (μmol.l/L) COLO 320	Cell proliferation (%) COLO 320 (72 hours)	*p*-Value
3.125	71.91<br>139.94<br>110.20	0.9399
6.25	55.16<br>44.64<br>55.42	0.0122
12.5	28.34<br>23.48<br>34.15	0.0008
25	16.48<br>14.93<br>20.41	0.0003

Table S3. Percentage of cell proliferation after 72-hour exposition of chalcone 1C in COLO 320, Human colorectal adenocarcinoma cells overexpressing ABCB1 trans-porter as determined by the MTT assay. Compared to the DMSO treated control group (set to 100% proliferation).

Concentration 1C (μmol/L) FHC	Cell proliferation (%) FHC (48 hours)	*p*-Value
5	108.21<br>95.04<br>96.32	>0.9999
25	112.39<br>96.22<br>103.03	0.991
50	114.61<br>95.64<br>103.22	0.9828
100	125.11<br>93.57<br>99.58	0.9423
150	119.80<br>92.07<br>89.38	>0.9999

Table S4. Percentage of cell proliferation after 48-hour exposition of chalcone 1C in FHC, Human normal fetal colon epithelial cells as determined by the SRB assay. Compared to the DMSO treated control group (set to 100% proliferation).

Concentration 1C (μmol/L) HT-29	Cell proliferation (%) HCT-29 (48 hours)	*p*-Value
1.5	101.64<br>99.47<br>85.43	0.9339
3	101.07<br>109.14<br>111.64	0.7076
5	94.50<br>111.78<br>97.25	0.9997
10	73.45<br>93.69<br>96.35	0.2837
50	40.28<br>31.76<br>28.10	<0.0001

Table S5. Percentage of cell proliferation after 48-hour exposition of chalcone 1C in HT-29, Human colorectal adenocarcinoma cells as determined by the SRB assay. Compared to the DMSO treated control group (set to 100% proliferation).

Transporter	MAF (%) COLO 205	MAF (%) COLO 320	*p*-Value
ABCB1	41.78<br>29.81<br>34.39	60.67<br>73.49<br>75.32	0.0049
ABCC1	65.95<br>38.66<br>46.84	58.42<br>84.74<br>80.17	0.1047
ABCG2	30.14<br>15.87<br>13.13	17.57<br>15.08<br>18.88	0.6808

Table S6. Multidrug Resistance Activity Factor (MAF) calculated from geometric means from 3 separate experiments. *p*-Value was calculated between MAF of COLO 205 and COLO 320.

Figure S1. Chemical structure of chalcone 1C (2E)-3-(acridin-9-yl)-1-(2.6-dimethoxyphenyl)prop-2-en-1-one (1C)

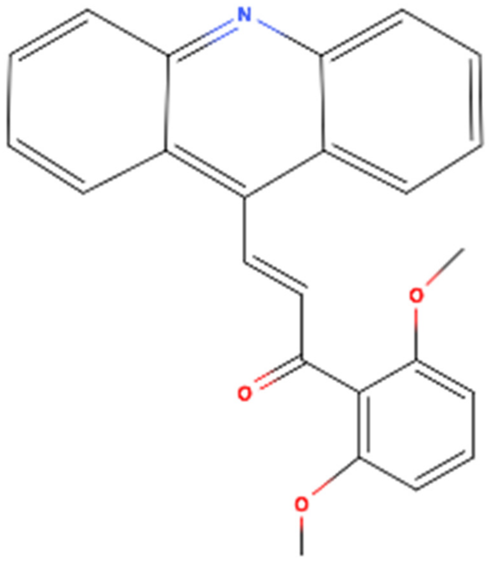

Supplement: Supplementary file 1 [file ijms-26-04138-s001.zip › ijms-3542109-supplementary.pdf]
